# Oxidative Stress and Inflammation as Targets for Novel Preventive and Therapeutic Approaches in Non-Communicable Diseases III

**DOI:** 10.3390/antiox13111404

**Published:** 2024-11-16

**Authors:** Chiara Nediani, Jessica Ruzzolini, Monica Dinu

**Affiliations:** 1 Department of Experimental and Clinical Biomedical Sciences “Mario Serio”, University of Florence, 50134 Florence, Italy; chiara.nediani@unifi.it (C.N.); jessica.ruzzolini@unifi.it (J.R.); 2 Department of Experimental and Clinical Medicine, University of Florence, 50134 Florence, Italy

Non-communicable diseases (NCDs), including cardiovascular diseases, diabetes, and neurodegenerative disorders, pose a significant global health challenge [1,2]. Central to their pathogenesis are two interlinked mechanisms: oxidative stress and inflammation [3,4]. Oxidative stress arises from the excessive production of reactive oxygen species (ROS), overwhelming the body’s antioxidant defenses, while inflammation is a key protective response triggered by tissue damage or infection. Extensive research indicates that these processes are not only pivotal in the onset of NCDs but also drive their progression [5,6]. This Special Issue compiles 17 articles that explore various dimensions of oxidative stress and inflammation, offering new insights into preventive and therapeutic strategies.

One area addressed is the role of oxidative stress and inflammation in renal diseases, a topic of growing interest in the literature [7,8,9]. Liu et al. [10] investigate the predictive value of interleukin-18 (IL-18) and superoxide dismutase 3 (SOD3) in patients with end-stage renal disease (ESRD). Their findings reveal that elevated IL-18 and reduced SOD3 levels are linked to a higher risk of kidney-related complications and mortality. Similarly, Khalaf et al. [11] identify the protective role of paraoxonase-1 (PON1), a hydrolytic enzyme, in chronic kidney disease (CKD). In PON1-deficient rats (SS-PON1 KO), increased renal injury—characterized by fibrosis, sclerosis, and acute tubular damage—was observed compared to control Dahl salt-sensitive rats. These findings suggest that modulating PON1 activity may represent a promising therapeutic target for mitigating inflammatory processes in CKD progression. In the context of acute kidney injury, Cheng et al. [12] highlight the potential of dexmedetomidine (DEX)-preconditioned adipose-derived stem cell microvesicles (DEX-MVs) in reducing renal ischemia/reperfusion (IR) injury. In a mouse model, DEX-MVs downregulated miR-122-5p-mediated oxidative stress and upregulated protective factors such as Bcl2 and erythropoietin, reducing tubular cell apoptosis and enhancing renal function. This approach offers a safer alternative to intravenous stem cell therapy by reducing immune rejection while enhancing cellular protection.

Mitochondrial dysfunction, a recurring theme in NCD progression, is especially notable for its role in generating oxidative stress and activating inflammatory pathways [13]. Monserrat-Mesquida et al. [14] describe how oxidative stress triggers key inflammatory pathways, including nuclear factor-kappa B (NF-κB) and mitogen-activated protein kinases (MAPKs), which are central to cardiovascular and neurodegenerative disease pathogenesis. These findings underscore the therapeutic potential of antioxidants, which neutralize ROS and modulate inflammatory signaling, offering promising strategies for managing multiple NCDs [15,16]. Manea et al. [17] further investigate oxidative stress in cardiovascular disease, identifying lysine-specific demethylase 1 (LSD1) as a potential therapeutic target. Their study demonstrates that inhibiting LSD1 in hypercholesterolemic mice reduces atherosclerotic lesions and oxidative stress by downregulating NADPH oxidase (Nox) subunits involved in ROS production, which in turn decreases inflammation and pro-inflammatory gene expression.

Endothelial dysfunction, a precursor to atherosclerosis, is tightly linked to oxidative stress and chronic inflammation [18]. The review by Higashi et al. [19] highlights how these processes reinforce one another, leading to cardiovascular complications. Oxidative stress, often driven by the activation of NADPH oxidase, xanthine oxidase, and mitochondrial dysfunction, promotes endothelial damage, while chronic inflammation sustains atherosclerosis progression. This interplay underscores the importance of targeting both oxidative stress and inflammation in cardiovascular disease management.

Alzheimer’s disease (AD) exemplifies the critical roles of oxidative stress and inflammation in neurodegenerative disorders. The accumulation of neurotoxic amyloid-beta (Aβ) peptides and neurofibrillary tangles induces neuroinflammation and synaptic dysfunction via oxidative stress pathways [20,21,22]. Natural compounds, such as polyphenols and vitamins, are gaining attention for their potential to slow AD progression [23,24,25]. Khan et al. [26] report that caffeic acid, a polyphenol found in various fruits and vegetables, protects against cognitive decline in AD model mice by reducing neuroinflammation and restoring neuronal function. Similarly, Shah et al. [27] review the roles of vitamins and minerals in AD, emphasizing their antioxidant and anti-inflammatory properties as well as their potential to influence amyloid precursor protein processing and blood–brain barrier integrity, thereby contributing to neuroprotection.

Oxidative stress and inflammation also significantly contribute to bone loss and osteoporosis, as discussed by Marcucci et al. [28]. They highlight the role of oxidative stress in inducing osteocyte apoptosis, which impairs bone remodeling. Natural antioxidants prevent this process by preserving osteocyte viability, promoting osteogenesis, and supporting bone formation, underscoring the potential of antioxidant supplementation to complement traditional antifracture therapies. Diet-derived antioxidants offer further benefits beyond isolated supplements [29].

Natural compounds are increasingly recognized for their broad therapeutic potential across various diseases [30,31,32,33,34]. Lien et al. [35] demonstrate the gastroprotective properties of *Anisomeles indica*, a traditional herb, in preventing aspirin-induced gastric ulcers. Their findings show that *A. indica* fractions enriched in ovatodiolide reduce inflammation and gastric acidity, offering a novel approach for treating NSAID-induced gastric injury. Peri et al. [36] explore the anticancer effects of oleocanthal, an extra-virgin olive oil extract, on chemotherapy-resistant gastric cancer cells. Oleocanthal reduces cell viability, inhibits colony formation, and induces apoptosis through ROS production, highlighting its potential against resistant cancer cells in combination with chemotherapy. Flavonoids have also been studied for their benefits in chronic disorders like inflammatory bowel disease [37,38,39]. Smeriglio et al. [40] report that citrus flavonoids, including hesperidin and hesperetin, exhibit strong antioxidant and anti-inflammatory effects in IL-1β-stimulated Caco-2 cells. Laudani et al. [41] summarize evidence on the cardioprotective effects of anthocyanins, a class of polyphenols. While clinical studies show that anthocyanin intake supports cardiovascular health by modulating gut microbiota and reducing inflammation [42,43,44], the variability in gut microbiota among individuals suggests the need for further research. Liao et al. [45] investigate the anti-inflammatory properties of kefir by comparing kefiran (KE) and kefir exopolysaccharides (KEPSs) in murine macrophages and transgenic mice. Kefir, a fermented milk product, has been widely studied for its health benefits. Research shows that microorganisms in yogurt starter cultures produce bioactive compounds, such as lactic acid, peptides, and bacteriocins, during milk fermentation, which contribute to kefir’s positive effects on nutrition and health, including immune support, enhanced digestive function, and potential benefits for conditions like hypertension, allergies, metabolic disorders, and heart disease [46,47,48]. Liao et al. [45] further demonstrate that KE and KEPS can reduce IL-6 secretion and inhibit NF-κB activation, supporting their potential to mitigate systemic inflammation.

Finally, several studies highlight the critical role of lifestyle interventions in managing and preventing metabolic disorders. Regular physical activity is widely acknowledged as essential for reducing oxidative stress and enhancing insulin sensitivity, two key factors implicated in these conditions [49,50]. Research shows that 150 min of physical activity per week, combined with a 7% weight reduction, can lower disease risk by 58% over three years. Physical activity alone improves insulin sensitivity and can reduce type 2 diabetes (T2DM) risk by 44% [51]. Piotrowska et al. [52] further demonstrate that regular exercise lowers pro-inflammatory cytokines, such as TNF-α and IL-6, while increasing anti-inflammatory mediators like IL-10, underscoring the role of exercise in diabetes management. Barrea et al. [53] advocate for incorporating bioactive compounds with antioxidant and anti-inflammatory properties, such as flavonoids and polyphenols, into the diet to enhance glycemic control and reduce inflammation in T2DM patients. Additionally, Colombini et al. [54] explore the impact of circadian rhythm disruptions on systemic inflammation, especially with age. Circadian rhythms are 24-hour cycles that govern vital physiological, metabolic, and endocrine processes, such as hormone secretion, body temperature, and the cell cycle, through the circadian clock system [55]. In this context, the concept of *inflammaging* underscores how age-related circadian disruptions exacerbate chronic inflammation [56]. Emerging research suggests that nutritional and pharmacological interventions may help counteract these disruptions, reducing inflammation and promoting healthier aging [57,58].

Together, these studies deepen our understanding of the role of oxidative stress and inflammation in NCDs. The Guest Editors of this Special Issue extend their gratitude to all contributing authors and reviewers for their invaluable insights and to the *Antioxidants* team for their continuous support. The integration of novel biomarkers, antioxidant therapies, and lifestyle interventions—such as exercise and personalized nutrition—presents exciting opportunities for the prevention and treatment of NCDs. Further research is essential to validate these approaches and develop targeted therapies to modulate oxidative stress and inflammation, ultimately improving health outcomes worldwide.
